# Review on SERS of Bacteria

**DOI:** 10.3390/bios7040051

**Published:** 2017-11-13

**Authors:** Pamela A. Mosier-Boss

**Affiliations:** Global Energy Corporation, 5101B Backlick Rd., Annandale, VA 22003, USA; pboss@san.rr.com; Tel.: +1-858-576-6415

**Keywords:** surface-enhanced Raman spectroscopy (SERS), bacteria, nanoparticles, secretions, cell envelope, principal component analysis (PCA)

## Abstract

Surface enhanced Raman spectroscopy (SERS) has been widely used for chemical detection. Moreover, the inherent richness of the spectral data has made SERS attractive for use in detecting biological materials, including bacteria. This review discusses methods that have been used to obtain SERS spectra of bacteria. The kinds of SERS substrates employed to obtain SERS spectra are discussed as well as how bacteria interact with silver and gold nanoparticles. The roll of capping agents on Ag/Au NPs in obtaining SERS spectra is examined as well as the interpretation of the spectral data.

## 1. Introduction

Similar to normal Raman spectroscopy, surface enhanced Raman spectroscopy (SERS) is an emission technique that involves inelastic scattering of incident laser energy resulting in spectral peaks, due to the vibrational modes of the molecule, that are frequency shifted from the incident energy. In the SERS technique, the Raman scattering is enhanced by molecules adsorbed on gold/silver roughened surfaces or nanostructures. Virtually all polyatomic species display a characteristic Raman/surface enhanced Raman spectroscopy (SERS) scattering spectrum. A recent review of the use of SERS for chemical detection discussed how advancements in nanostructure fabrication have dramatically advanced SERS capabilities as a powerful chemical sensing platform [1]. This same review also indicated that, in addition to advances in nanoscience, the field of photonics saw the development of low-power, compact, robust, inexpensive Raman systems that could be deployed in the field. This convergence of technologies (photonics and nanoscience) has led to a greater use of the SERS technique to detect not only chemical species, but biological analytes as well. In this communication, the use of SERS to obtain vibrational spectra of bacteria is discussed. The main advantages of SERS for this purpose are the sensitivity of the technique and the high resolution of the spectra. The later makes simultaneous multicomponent analysis possible, which is particularly important given the complexity of bacterial cells. The methods used to obtain SERS spectra of bacteria are discussed as well as the kinds of substrates used. Other pertinent areas of discussion include the roll capping agents play on obtaining SERS spectra, speciation, and interpretation of the spectral data.

## 2. Results and Discussion

### 2.1. Bacteria and the Antimicrobial Properties of Ag/Au Nanoparticles (NPs)

What is observed in SERS spectra is determined by the molecular species that are in proximity to the Ag/Au nanoparticles. This, in turn, is determined by the manner in which bacteria and Ag/Au nanoparticles interact. Therefore, to be able to interpret SERS spectra obtained for bacteria, it is imperative to know what bacteria are comprised of and how Ag/Au NPs interact with them. To this end, a brief tutorial on bacterial structure is provided. Metals, including silver and gold, have been known to have antimicrobial properties since ancient times [2]. How these metals interact with bacteria influences not only their antimicrobial activities, but also dictates what is observed in the SERS spectra. Consequently, a discussion of the antimicrobial activity of silver is included in this review.

#### 2.1.1. Structure of Bacteria

**Figure 1:**
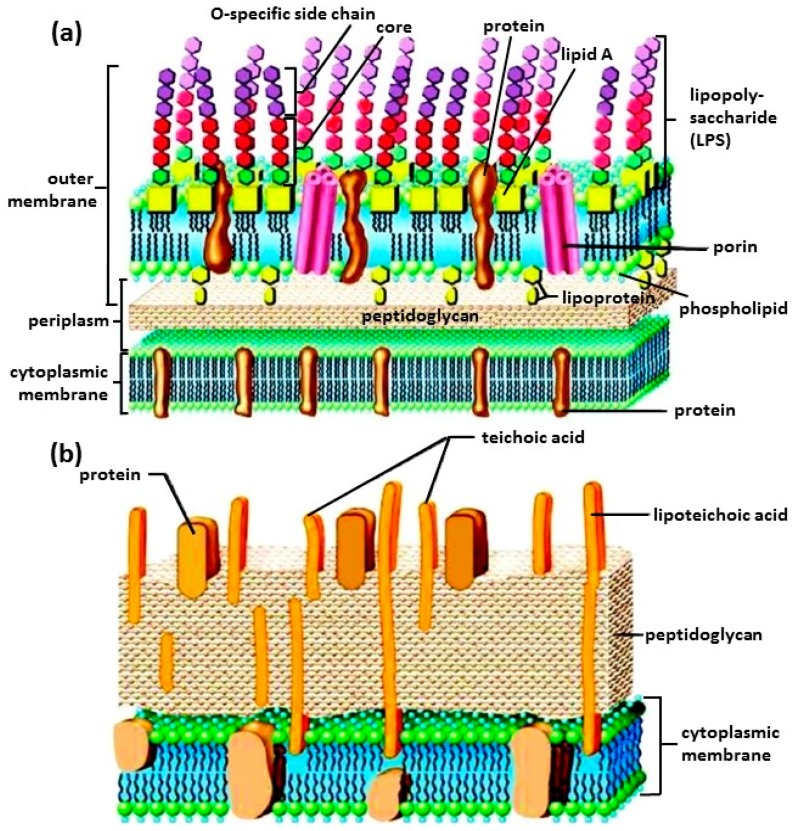
A cartoon view of the membranes of: (**a**) Gram-negative bacterium; and (**b**) Gram-positive bacterium. The membranes of Gram-negative bacteria are composed of two layers: the outer membrane rich in lipopolysaccharide (LPS) and the inner membrane rich in anionic phosphatidylglycerols (PG). Gram-positive bacteria have a cell wall consisting of lipoteichoic acid and peptidoglycan and a cytoplasmic membrane. Reproduced with permission from Elsevier [4].

Bacteria are prokaryotic microorganisms, meaning they have no membrane-bound organelles such as a nucleus or mitochondria [3]. They are typically a few micrometers in size and have shapes ranging from spheres to rods and spirals. Instead of a nucleus, the genetic material is typically a single circular bacterial chromosome of DNA located in the cytoplasm in an irregularly shaped body called the nucleoid. The bacterial cell is surrounded by a cytoplasmic membrane. Outside the cytoplasmic membrane is the cell wall. Together, the cell wall and the cytoplasmic membrane make up the cell envelope. Bacterial cell envelopes fall into two major categories: a Gram-positive type and a Gram-negative type. Schematics of both Gram-positive and Gram-negative cell envelopes are shown in Figure 1 [4]. Gram staining differentiates bacteria by the chemical and physical properties of their cell walls by detecting peptidoglycan, which is present in the cell wall [5]. As shown in Figure 1, Gram-positive bacteria have a thick peptidoglycan layer while Gram-negative bacteria have a thin layer. In general, 90% of the Gram-positive cell wall is comprised of peptidoglycan [6]. In contrast, the Gram-negative cell wall is 10–20% peptidoglycan. As a result, during Gram staining, Gram-positive bacteria retain the crystal violet dye and are stained violet [5]; Gram-negative bacteria do not, and are stained pink.

The bacterial cell wall provides structural integrity to the cell [6] Peptidoglycan is a polymer consisting of sugars and amino acids that forms a porous, mesh-like layer outside the cell membrane. As shown in Figure 1b, polyalcohols, known as teichoic acids, are imbedded in the Gram-positive cell wall. Some of these polyalcohols are lipid-linked to form lipoteichoic acids. These lipoteichoic acids are covalently linked to lipids within the cell membrane and are, therefore, responsible for linking peptidoglycan to the cell wall. The Gram-negative bacterial cell envelope is a multilayered cell surface consisting of an outer membrane, peptidoglycan layer in the periplasmic space, and a cell membrane, Figure 1a [5,6]. The outermost layer of the Gram-negative wall is the outer membrane, which consists of three regions: the most conservative lipid A, core polysaccharide, and O-specific antigen [4]. Porins, or pore-like structures, are present in the outer membrane which allow the diffusion of nutrients into the cell and the dissemination of wastes [7]. The periplasmic space is between the outer membrane and the inner cytoplasmic membrane [7]. It is filled with a gel-like matrix called the periplasma, which contains binding proteins for amino acids, sugars, vitamins, ions, as well as degradative and detoxifying enzymes. In both Gram-positive and Gram-negative bacteria, the cell membrane is a phospholipid bilayer with embedded proteins that controls the movement of materials in and out of the cell [8].

In both eukaryotes and prokaryotes, adenosine triphosphate (ATP) is used to store and transport chemical energy within cells. In eukaryotic cells, ATP production occurs in the mitochondria. However, prokaryotes, such as bacteria, do not have mitochondria. Instead, in bacteria, the ATPase and the electron transport chain are located *inside* the cytoplasmic membrane between the hydrophobic tails of the phospholipid membrane inner and outer walls [9]. Breakdown of sugar and other food causes the positively charged protons on the *outside* of the membrane to accumulate to a much higher concentration than they are on the membrane *inside*. This creates an excess positive charge on the outside of the membrane and a relatively negative charge on the inside. The result of this charge difference is a dissociation of H_2_O molecules into H^+^ and OH^−^ ions. The H^+^ ions that are produced are then transported outside of the cell and the OH^−^ ions remain on the inside. This results in a potential energy gradient similar to that produced by charging a flashlight battery. The force the potential energy gradient produces is called a proton motive force that can accomplish a variety of cell tasks including converting ADP into ATP. The cytoplasmic membrane takes care of not only the cell’s energy conversion needs, but also nutrient processing, synthesizing of structural macromolecules, and secretion of the many enzymes needed for life. As shall be seen, all this activity becomes relevant when interpreting the SERS spectral data obtained for bacteria.

Some bacteria may have a capsule that is more external than the cell envelope. This capsule is not shown in Figure 1. Bacterial capsules are composed of high-molecular-weight polysaccharides and/or polypeptides, and are associated with virulence and biofilm formation [10]. The capsule protects the cell from desiccation and engulfment by macrophages and prevents attachment of bacteriophages on the cell surface [11].

#### 2.1.2. Antimicrobial Activity of Ag/Au NPs: Interaction of Ag/Au NPs with Bacteria

Silver and gold exhibit antimicrobial properties [2,12,13]. Before the beginning of antibiotics therapy, silver was used for its antiseptic activity, specifically for the treatment of open wounds and burns [12]. The most critical physico-chemical parameters that affect the antimicrobial potential of AgNPs include surface chemistry and charge, size, size distribution, shape, particle composition and morphology, coating/capping, concentration, agglomeration, dissolution rate, and type of reducing agents used for synthesis [14,15]. One study synthesized Ag NPs in sizes ranging from 5 to 100 nm, using the same protocol [16]. This study showed that, while all Ag NPs were highly toxic to the bacterial strains investigated, their antibacterial efficacy increased with lowering particle size. Another study showed that smaller sized, plate shaped Ag NPs presented higher antimicrobial activity than larger sized cubic and spherical Ag NPs [17]. The antibacterial activity of silver nanoparticles depended upon how they were fabricated. Borate-silver nanoparticles were inactive against *Salmonella*, whereas citrate-silver and polyvinylpyrrolidone (PVP)-silver exhibited antibacterial activity [18]. Ag NPs produced by the sodium borohydride reduction method are uncapped [19] while citrate and PVP are capping agents commonly used to stabilize Ag colloids [18]. Microscopic examination of *E. coli* treated with Ag NPs showed that borohydride generated Ag NPs were not effective in retarding growth of the bacterial cells [20]. In contrast, citrate generated Ag NPs not only effectively retarded the growth of the cells but also greatly reduced their size. The antibacterial action of pure Ag NPs were enhanced upon dispersion in surfactants such as sodium dodecyl sulfate (SDS), sodium dodecyl benzene sulfonate (SDBS), triton X-100 (TX-100), and polysorbate 80 (Tween 80) [21]. Likewise, an increase in antibacterial activity of beta-cyclodextrin (β-CD)-capped Ag NPs compared with their uncapped equivalents was observed [22]. This activity increased as the concentration of β-CD increased. These observations indicate that capping agents enhance the antibacterial activity of the Ag NPs.

**Figure 2:**
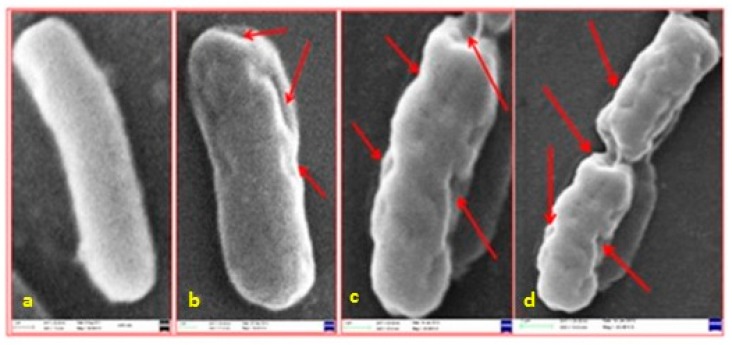
SEM photomicrographs of *E. coli*: (**a**) control; and (**b**) 10; (**c**) 20; and (**d**) 30 μg·mL^−1^ gum arabic capped Ag NPs (arrows indicate depressions and extensive damage of bacterial cells). Reproduced with permission from John Wiley and Sons [23].

**Figure 3:**
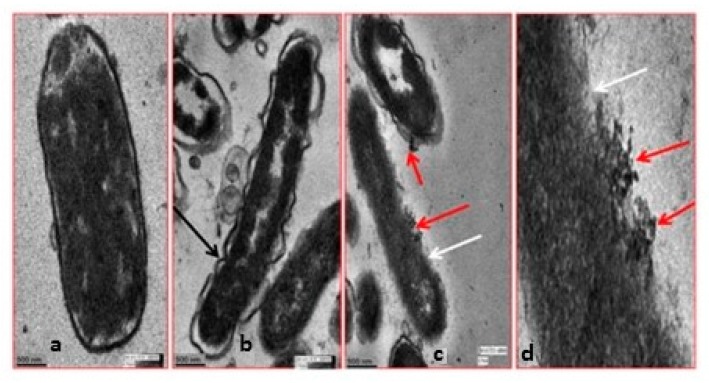
HR-TEM images of *E. coli* cells: (**a**) untreated; (**b**,**c**) treated with gum arabic Ag NPs; and (**d**) enlarged view of the membrane of (**c**). Red arrow indicates Ag NPs attachment on membrane and black and white arrows show partially damaged membranes at various sites. Reproduced with permission from John Wiley and Sons [23].

Figure 2 and Figure 3 show scanning electron microscopy (SEM) and high-resolution transmission electron microscopy (HR-TEM) images, respectively, obtained for *E. coli* cells that had been exposed to gum arabic coated Ag NPs [23]. In Figure 2a, native *E. coli* cells were rod-shaped and smooth. There was no observable damage on the cell surface. When treated with gum arabic coated Ag NPs, the cells became severely damaged, Figure 2b–d. Multiple indentations and depressions were observed on the cells. It can be seen that the greater the concentration of Ag NPs, the greater the extent of damage. The HR-TEM image of the control *E. coli* cell, Figure 3a, showed a multilayered cell surface consisting of an outer membrane, a peptidoglycan layer in the periplasmic space, and a cytoplasmic membrane. HR-TEM images of cells exposed to gum arabic coated Ag NPs, Figure 3b,c, showed cracked and ruptured cells. Electron-dense silver particles were observed around damaged bacterial cells. The damaged cells showed either localized or complete separation of the cell membrane from the cell wall. The damaged cells also exhibited electron-translucent cytoplasm as well as cellular disruption. In Figure 3c,d, the HR-TEM micrographs show the accumulation of silver nanoparticles in the membrane as well as inside the cell.

**Figure 4:**
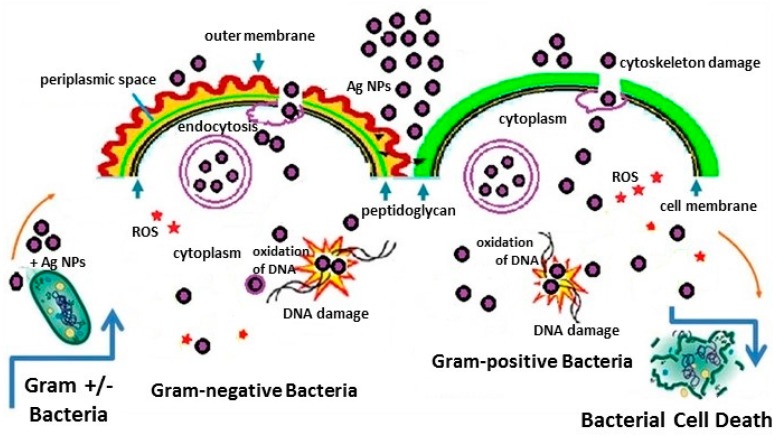
Schematic diagram of bactericidal activity of Ag NPs on Gram-positive and Gram-negative bacteria. Reproduced with permission from Springer [24]. Ag NPs adhesion to microbial cells, penetration inside the cells, ROS and free radical generation, and modulation of microbial signal transduction pathways have been recognized as the most prominent modes of antimicrobial action [13].

Figure 4 summarizes the different pathways of bactericidal activity of Ag NPs on Gram-positive and Gram-negative bacteria [24]. The HR-TEM images indicate that the Ag NPs attach to the surface of the bacterial cell wall first, then permeate the cell membrane and enter the cell interior [23]. As discussed above, these interactions were facilitated by the capping agents present on the surface of the Ag NPs. The Ag NPs attached to the cell membrane release Ag ions that interact with sulfur and phosphorous containing compounds, such as proteins and DNA, on and inside the cell membranes [25]. These Ag ions may inhibit DNA replication resulting in a loss of cell viability which ultimately leads to cell death. Once inside the bacterial cells, Ag NPs can have a sustained release of Ag ions that can then interact with thiol groups present in enzymes such a nicotinamide adenine dinucleotide (NADH) dehydrogenases and disrupt the respiratory chain. The formation of free radicals by Ag NPs induces oxidative stress that can also lead to cell death. Consequently, the major processes underlying the antibacterial effects of metal nanoparticles are: (1) disruption of the bacterial cell membrane causing leakage of the intracellular contents; (2) generation of reactive oxygen species (ROS) that interact with the cell wall and membrane causing damage to the cell membrane; (3) penetration of the bacterial cell membrane; and (4) induction of intracellular antibacterial effects, including interactions with DNA and proteins [23,25,26].

### 2.2. Methods and Substrates Used to Obtain SERS Spectra of Bacteria

Bacterial species are grown in a nutrient medium. For microorganisms, the nutrient medium contains a carbon source such as glucose, water, various salts, and a source of amino acids and nitrogen (e.g., beef broth, yeast extract). As such, the constituents of the nutrient medium will exhibit SERS spectral peaks that will overlap with the bacterial peaks [27]. Consequently, to remove residual nutrient medium, the cells need to be washed. It has been shown that removal of cell growth culture can be accomplished by doing a minimum of three standard water-washing/centrifugation cycles [28]. While many strains of bacteria are quite hardy and can withstand suspension in water without apparent impacts due to osmotic imbalances, some strains of bacteria are sensitive to changes in osmolarity and lyse. To prevent the cells from lysing, they need to be washed with a buffer. Of the buffers examined in a previous investigation [29], borate was the only one that did not suppress the SERS effect. Borate buffer also did not exhibit an appreciable SERS spectrum.

In general, three approaches have been used to obtain SERS spectra of bacteria. One approach is to form colloidal silver directly on, or inside, the individual bacteria. In the second approach, the bacteria are placed directly on a SERS active surface. For the third approach, bacteria and colloid are mixed together and the mixture is placed upon a flat surface. A description of these methods and the spectral results are discussed.

#### 2.2.1. SERS Spectra Obtained by Forming Colloidal Silver/Gold on/inside the Bacteria

**Figure 5:**
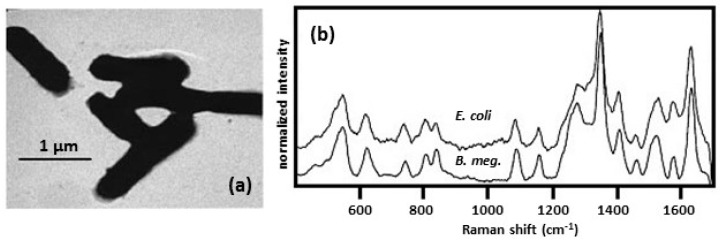
(**a**) TEM image of *E. coli* with a wall colloid [30]. Reproduced with permission from the American Chemical Society. (**b**) SERS spectra obtained for *E. coli* and *B. megaterium* coated with silver [31]. Spectra were obtained using 514.5 nm excitation. Reproduced with permission from Elsevier.

One approach to obtain SERS spectra of bacteria is to form colloidal silver or gold directly on the individual bacteria. The most common method used was to soak the bacteria in a solution of sodium borohydride [30,31,32,33,34,35]. The cells were then centrifuged and rinsed with water to remove excess sodium borohydride. The bacteria were then resuspended in either a solution of silver nitrate or chloroauric acid (HAuCl_4_). The result was a colloid that was concentrated on the cell wall, where the incoming diffusion of the metal ions met the outgoing diffusion front of the reductant and where the wall served as an efficient nucleation site [34]. As shown in Figure 5a, the silver/gold metal formed a rough film over the exterior of the bacterial cells and the bacteria can be seen to be sticking together. Since the shapes of the bacteria were still discernable, the thickness of the Ag film encapsulating the bacteria was estimated to be ~0.05 μm. Samples of the bacteria were placed on a flat surface, such as a glass slide or Al SEM sample stub, and allowed to dry before SERS spectra were collected. Figure 5b shows SERS spectra obtained for *E. coli* and *B. megaterium* [31]. While *E. coli* is a Gram-negative bacterium, *B. megaterium* is Gram-positive. As shown in Figure 1, the structures of the cell envelopes for Gram-positive and Gram-negative bacteria are very different. Despite these differences, the SERS spectra look very similar suggesting that the SERS is dominated by peaks/bands from a small number of molecules present in the cell walls. Similar observations were made comparing SERS spectra of *E. coli* and *B. subtilis* [33]. Earlier, a SERS study was done on a photosynthetic bacterium [36]. In this study, the wall from the interior and exterior sides was spread on a silver electrode in an electrochemical cell. The SERS spectra were similar and were assigned to flavin adenine dinucleotide (FAD). The spectral features observed in the electrochemical study [36] were practically identical to those observed for the complete, intact cells coated with silver colloid [30,31,32,33,34]. This indicated that FAD derivatives are incorporated into the cell wall. HPLC, followed by UV-VIS spectroscopy and LC-MS, showed that *Shewanella* species secreted flavins [37]. This study further showed that, while FAD was the predominant intracellular flavin, it was not released by live cells.

**Figure 6:**
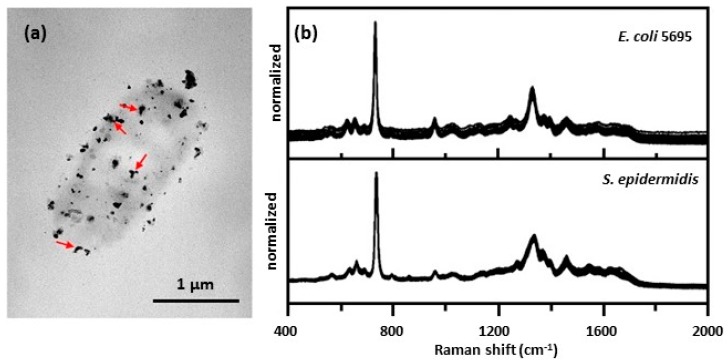
(**a**) TEM image of *E. coli* 1116 showing the colloid deposits that formed on the cell wall (bacteria(H_2_O)@AgNP) [38]. Red arrows indicate Ag composites that have large, elongated structures. (**b**) SERS spectra obtained for *E. coli* 5695 and *S. epidermidis* in contact with clusters of Ag NPs [38]. Spectra were obtained for 15 different batches of samples using 633 nm excitation. Reproduced with permission from the American Chemical Society.

Zhou et al. [38,39,40] prepared an external colloid on bacteria using a different methodology. In their procedure, they added a silver nitrate solution to a sample of bacteria. After 5 min, they then added a solution of hydroxylamine hydrochloride to reduce the silver ions adsorbed on the bacterial cell walls. A sample of the suspension was pipetted onto a glass slide, or poly-l-lysine coated glass slide, prior to obtaining SERS spectra. Poly-l-lysine coated glass slides had to be used to immobilize Gram-positive bacteria [39,40]. Figure 6a shows a TEM image of an *E. coli* cell showing that the Ag NPs had formed on the cell wall and that they were in intimate contact with the cell wall [38]. While the borohydride preparation described above created a Ag/Au coating that completely encapsulated the bacterial cells, (Figure 5a) [30,31], clusters of Ag NPs were observed to be in contact with the cell wall, Figure 6a, and the cell was not fully encapsulated [38]. Figure 6b shows SERS spectra obtained for *E. coli* (Gram-negative) and *S. epidermidis* (Gram-positive). Both sets of spectra were very similar and greatly resemble that obtained for adenosine monophosphate (AMP) on borohydride-generated Ag NPs after adding acetic acid to pH 5.0 [41]. The large peak at 732 cm^−1^ is assigned to the purine ring-breathing mode. This peak is greatly enhanced compared to the normal Raman spectrum of AMP. The large enhancement of this vibrational mode indicates that adsorption of AMP onto the Ag surface is through the purine moiety. Similar results were obtained using this methodology to obtain SERS spectra of Gram-positive bacteria [42].

Flavins are extremely important redox coenzymes used in a variety of biochemical processes [32]. The flavin group is a tricyclic heterocycle isoalloxazine that is capable of undergoing oxidation-reduction reactions and can accept either one electron in a two-step process or two electrons at once. Flavin compounds found in practically all bacteria include riboflavin (RF), vitamin B2, flavin adenine mononucleotide (FMN), and FAD. These flavins are found in the plasma membrane as part of the flavoprotein complex. The fact that the SERS spectra obtained using the external wall colloid method are dominated by spectral features due to flavins suggests that the isoalloxazine and, when present, adenine moieties serve as highly effective nucleation enters for the formation of the Ag NPs. The silver ions would have a tendency to preferentially accumulate near flavins and other adenine containing molecules. This will cause the Ag NPs to form where these compounds are present. There is evidence of such silver ion-flavin complexes in the literature. A crystal structure of a silver ion-riboflavin complex has been reported [43]. A spectrophotometric method was used to obtain a stability constant of log 5.82 (at pH 7) for the silver ion-riboflavin complex [44]. Resonance Raman was used to study the silver ion complex with FMN [45].

**Figure 7:**
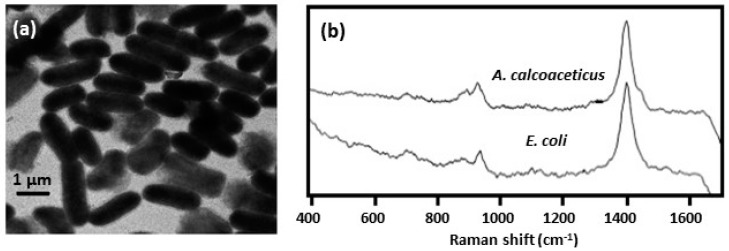
(**a**) TEM image of *E. coli* infused with Ag colloid [34]. (**b**) SERS spectra of *E. coli* and *A. calcoaceticus* infused with silver colloid [34]. Spectra were obtained using 514.5 nm excitation. Reproduced with permission from John Wiley and Sons.

To prepare Ag colloid inside the bacterial cells, the cells were first washed in silver nitrate solution [30,32,34]. After washing off the excess silver nitrate, sodium borohydride solution was added. As shown in Figure 7a, a uniform colloid formed predominantly inside the bacteria [30]. The bacteria remain mostly separated and there was no evidence of bridging between them. Figure 7b shows SERS spectra of bacteria obtained using the internal colloid [32,34]. The spectra were generally much weaker than those observed for the silver-coated bacteria, Figure 5b. Spectral features were also rather sparse and flavin spectral signatures were absent. The peaks at around 1400 and 930 cm^−1^ were assigned to carboxylate stretching and were attributed to aspartic and glutamic acids.

As a simple guide for the reader, the SERS spectra of bacteria obtained by forming colloids on/inside the bacteria are summarized in Table 1.

#### 2.2.2. SERS Spectra Obtained by Placing Bacteria Directly on a SERS-Active Surface

In the second approach used to obtain SERS spectra of bacteria, bacteria are placed on the surface of a SERS-active substrate [46,47,48,49,50,51,52,53,54,55,56,57,58,59,60]. Substrates used can be simple, such as roughened gold coated glass slides [43]. The glass slides were commercially available from EMP Corp. Cell suspensions of *Anthrobacter* strains were diluted with water and spotted on the surface of the substrate. Spectra were obtained when the samples had dried. These spectra were used to differentiate between fourteen different strains of *Anthrobacter*. Other substrates were more complex and have been used to monitor germination of bacterial spores [47] and quorum sensing (QS) between bacteria [48,49,61] in addition to bacterial detection [50,51,52,54,55,56,57,58,59].

**Figure 8:**
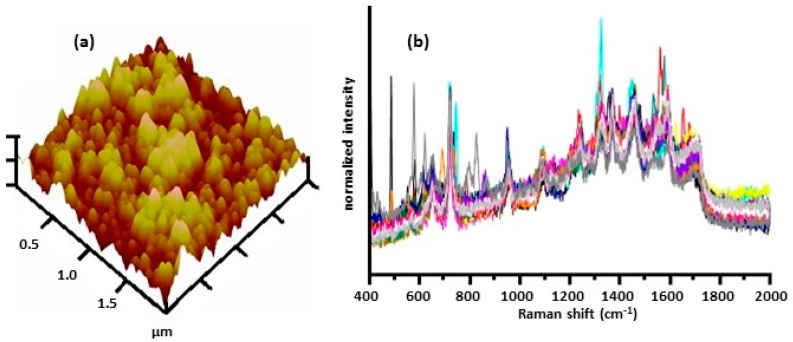
(**a**) AFM image of nanostructured PPX-Cl film deposited with 60 nm Au film [50]. (**b**) SERS spectra collected for 25 random cells on 1 mm^2^ area showing a highly reproducible fingerprint for *E. coli* [50]. Spectra were obtained using 785 nm excitation. Reproduced with permission from John Wiley and Sons.

Malvadkar et al. [50] prepared SERS substrates by depositing nanostructured films of poly(chloro-p-xylylene), PPX-Cl, on an allyl functionalized silicon wafer using oblique angle vapor deposition polymerization under low-vacuum conditions. A thin film of gold was then deposited onto the PPX-Cl surface using thermal evaporation to create the SERS-active surface. The nanostructure consisted of a parallel assembly of nanowire arrays having a diameter of 150 nm. Figure 8a shows an atomic force microscope (AFM) image of the surface after a gold layer was deposited on the nanostructured PPX-Cl film. An inoculation loop was used to place a sample of a bacterial suspension onto the SERS surface. Spectra were obtained using a Raman microscope. Figure 8b shows SERS spectra obtained for 25 separate *E. coli* cells. Kamińska et al. [51] prepared Ag/Au-coated, nucleopore track-etched polycarbonate membranes that allowed simultaneous filtration of cerebrospinal fluid (CSF) and immobilization of CSF components. Samples were filtered directly onto the surface of the Ag/Au-coated membranes. Using these SERS substrates, they obtained spectra of bacterial meningitis pathogens.

Lin et al. [52] prepared a gold nanoparticle embedded mesoporous silica by mixing borohydride generated Ag NPs-gelatin solution with an acidified silicate solution which was then calcinated at 600 °C for 6 h. A filter-like, SERS-active substrate was prepared by compressing the resultant powder in a stainless mold. Samples of *S. aureus* in water were dropped directly onto the surface of the substrate. The resultant SERS spectra were dominated by peaks at 1158 and 1520 cm^−1^, which were attributed to β-carotene on the cell wall of *S. aureus*. Wang et al. [53] prepared arrays of Ag NPs grown in porous anodic aluminum oxide (AAO) nanochannels with a precisely controlled variation of interparticle gaps between 5 and 25 nm. They used these substrates to obtain SERS spectra of various Gram-positive and Gram-negative bacteria [54,55]. They also obtained SERS spectra of bacteria at different growth phases which will be discussed below.

**Figure 9:**
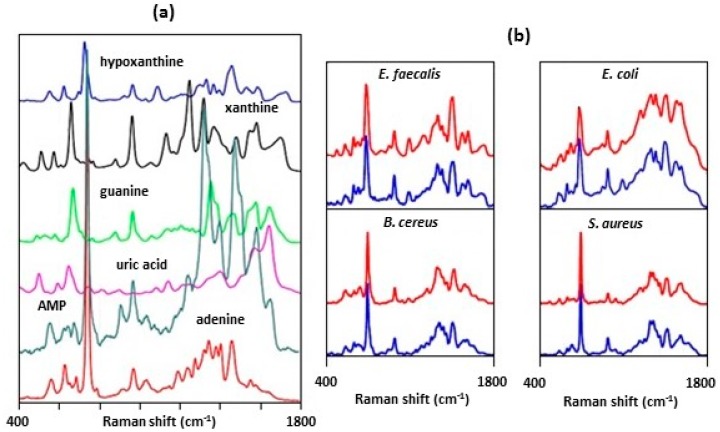
(**a**) SERS spectra of 20 μM aqueous solutions of the indicated purine components of bacterial SERS spectra. The spectra have been offset for viewing and are normalized to the maximum peak intensity of the 20 μM adenine solution [57]. (**b**) Comparison of the SERS spectra of cells (blue) for four bacterial strains and their corresponding enriched supernatant (red) [57]. Spectra of cells and supernatant are offset for better viewing. Spectra were obtained using 785 nm excitation. Reproduced with permission from Springer.

Premasiri et al. [56,57,58] used gold nanoparticle SiO_2_ substrates to obtain SERS spectra of bacteria. The aggregated gold nanoparticle coated SiO_2_ matrix was produced by a multistep in-situ growth procedure. A gold ion doped sol-gel was formed by the hydrolysis of tetramethoxysilane in an acidic methanol solution of HAuCl_4_. A sodium borohydride solution was used to reduce the gold ions to form aggregates of monodisperse, nano-sized gold spheres. The gold-impregnated sol gel was then placed on a glass chip and allowed to cure. To obtain SERS spectra, an inoculation loop was used to place a sample of a bacterial suspension onto the SERS surface. SERS spectra were obtained using a Raman microscope. They showed that the dominant molecular species contributing to the SERS spectra were the metabolites of purine degradation: adenine, hypoxanthine, guanine, uric acid, and AMP [57]. The SERS spectra of these metabolites are shown in Figure 9a. The blue SERS spectra shown in Figure 9b were for cells of four species of bacteria. These spectra are a linear combination of the SERS spectra of the metabolites shown in Figure 9a. For example, the SERS spectrum of *E. faecalis* has the following composition: 21% adenine, 61% hypoxanthine, 10% xanthine, and 8% guanine. These results showed that the Au NPs have a strong affinity for these compounds and preferentially interact with them. Purine compounds play important roles in many electron transfer reactions and are present on the interior of the bacterial cell wall [38,62]. To interact and bind with the Au NPs on the SERS substrate, these compounds need to be released, or secreted, from the cell. To demonstrate that these compounds were being secreted, Premasiri et al. [57] collected the supernatant created from washing the cells. They filtered the supernatant through a 0.22 μm filter to remove any residual bacterial cells. The supernatant was placed on a SERS substrate and are the red spectra shown in Figure 9b. As can be seen, the supernatant spectra are nearly identical to their corresponding bacterial cell SERS spectra.

Efforts have been made to immobilize [59] or isolate bacteria from a matrix [60] prior to detection by SERS. Substrates comprised of Ag dendrites, for SERS enhancement, were modified with 4-mercaptophenylboronic acid, which was used to capture the bacterial cells [59]. Capture efficiency for *S. enterica* was 84.92 ± 3.25% at 10^6^ cfu/mL and as high as 99.65 ± 3.58% at 10^3^ cfu/mL. Immunomagnetic beads for bacterial separation was combined with optical detection by SERS [60]. Antibody-coated magnetic beads were used to capture *L. innocua*. A neodymium magnet was then used to concentrate the beads, and their captured bacteria, onto the surface of a SERS-active chip comprised of Au NPs.

Placing bacteria on top of SERS substrates will not provide information on the bacterial cell membrane. This is because the immobilized Ag/Au NPs of these substrates cannot partition through the outer polysaccharide layers of the cell envelope, described in Figure 1, to bind to the cell membrane. However, these kinds of substrates can be used to study the metabolic degradation pathways, as shown by Premasiri et al. [57]. These substrates are also ideal for monitoring quorum-sensing in bacteria. Bodelón et al. [48] designed nanostructured SERS substrates for the in-situ, label-free detection of a QS signaling metabolite in growing *P. aeruginosa* biofilms and microcolonies. *P. aeruginosa* secrete a variety of redox-active phenazine compounds, including pyocyanin [63]. Besides being responsible for the blue-green color characteristic of *Pseudomonas* species, pyocyanine is both a virulence factor and a quorum sensing signaling molecule. Bodelón et al. [48] used hybrid materials comprised of a SERS-active component within a porous matrix that allowed diffusion of small molecules for detection and imaging of pyocyanine in biofilms and microcolonies of *P. aeruginosa*. They used microporous poly-*N*-isopropylacrylamide (pNIPAM) hydrogels loaded with Au nanorods (Au@pNIPAM) to detect pyocyanine homogeneously in both colonized and non-colonized regions of the substrate. Mesostructured Au@TiO_2_ substrates bearing a mesoporous TiO_2_ thin film over a sub-monolayer of Au nanosperes restricted pyocyanine detection to biofilm-colonized surfaces with a spatial resolution of ~20 μm. They also used a mesoporous silica-coated micropatterned supercrystal arrays of Au nanorods (Au@SiO_2_) to detect pyocyanine expression at early stages of biofilm formation. Polisetti et al. [61] used SERS imaging to do in situ spatial/temporal mapping of pyocyanine in communities of *P. aeruginosa*. They found that production of pyocyanine was dependent on the growth carbon source and on the specific strain of *P. aeruginosa*.

Schkolnik et al. [49] used SERS to follow the development and chemical composition of *S. oneidensis* biofilms formed at an Ag/AgCl solid interface. As *S. oneidensis* anaerobically colonized an Ag/AgCl solid interface, it precipitated silver nanoparticles. These Ag NPs adsorbed the molecules secreted by the bacteria during biofilm formation. This resulted in the in situ chemical mapping of the biofilm as it developed over time making it possible to monitor the distribution of cytochromes, polysaccharides, reduced and oxidized flavins, and phosphate in the undisturbed biofilm both spatially and temporally.

This methodology also provided a means of measuring the kinetics of germination of *B. subtilis* spores in situ by following the Raman peak at 1010 cm^−1^ characteristic of dipicolinic acid (DPA) that is released during germination. Germination of *B. subtilis* spores was measured by placing spores on a SERS substrate comprised of Ag NPs immobilized on a glass slide with a poly(diallyldimethylammonium chloride) (PDDA) coated Ag mirror film [47]. A solution with the germinant (l-alanine) was added and a blank slide was placed over the spores and solution. The sandwiched slides were immersed into a small preheated vial containing ~1 mL of water that was fitted into a large aluminum block with a heating element. Before germination and the addition of the germinating solution, the SERS spectrum was relatively featureless and showed two broad peaks at 589 and 794 cm^−1^ due to PDDA. These peaks remained constant throughout the experiments and were used as an internal standard. With the addition of the germinating solution, new bands at 847 and 942 cm^−1^, due to l-alanine, were observed in the spectrum. During germination, additional peaks were observed to grow in, including one at 1010 cm^−1^. This peak progressively increased as the germination proceeded and was attributed to the release of DPA from the core of the spores and adsorbing onto the Ag NPs immobilized on the Ag mirror film.

As a simple guide for the reader, the SERS spectra of bacteria obtained by placing bacteria on a SERS-active surface are summarized in Table 2. For this approach to work, the nanoparticles need to be uncapped. If capped, the SERS spectra will be dominated by the SERS spectrum of the coating [29].

#### 2.2.3. SERS Spectra Obtained by Mixing Colloids with Bacterial Suspensions

In the third approach used to obtain SERS spectra of bacteria, bacteria and colloid are mixed together [38,64,65,66,67,68,69,70,71,72,73,74,75,76]. Some researchers would obtain SERS spectra of the bacteria-colloid suspension. Others would spot the mixture onto a flat surface and obtain spectra once the sample had dried. Kahraman et al. [70] developed a convective assembly technique to deposit bacteria and citrate-generated silver nanoparticles on a glass slide as a thin film. The assembly process assured a homogeneous distribution of the bacteria-Ag NP mixture. In this process, a glass slide was attached to a moving stage. A mixture of bacteria and silver nanoparticles was spotted on the slide. A second, fixed slide was placed in contact with the mixture in such a way that the angle between the two slides was about 24° to create a meniscus. The glass slide, attached to the moving stage, was then moved forward at a rate of 1.0 μm/s. This spread the sample out into a thin film. SERS spectra were obtained after the sample had dried. Filtering the bacteria-Ag NP mixture thorough a 0.02 μm pore size, ceramic filter was another method developed to assure a homogeneous distribution of the bacteria-Ag NP mixture on a surface [76]. As will be shown, different spectra are obtained when using capped and uncapped nanoparticles. Table 3 summarizes SERS spectra obtained for bacteria using this methodology.

**Figure 10:**
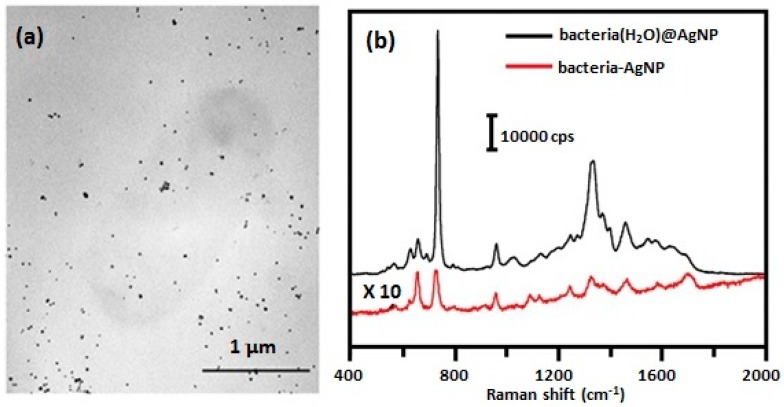
(**a**) TEM image of *E. coli* 1116 mixed with Ag NPs and 0.01 M NaCl (bacteria-AgNP) [38]. (**b**) SERS spectra obtained for *E. coli* 1116 obtained by two different methods: external colloid formation, bacteria(H_2_O)@AgNP, and mixing bacteria with Ag colloid, bacteria-AgNP [38]. Spectra were obtained using 633 nm excitation. Each sample was measured three times. Reproduced with permission from the American Chemical Society.

Besides obtaining SERS spectra of *E. coli* by forming an external colloid directly on the bacteria, Figure 6, Zhou et al. [38,64] also obtained spectra by mixing the bacteria with Ag NPs. The Ag NPs were prepared by reducing AgNO_3_ with hydroxylamine hydrochloride. The resultant silver nanoparticles were uncapped, or uncoated, and did not exhibit a Raman signature of their own [77]. TEM images of *E. coli* mixed with Ag NPs prepared by hydroxylamine hydrochloride reduction showed that the Ag NPs were randomly distributed and did not aggregate on the surface of the bacterial cells, Figure 10a. As was discussed above, uncapped Ag NPs do not exhibit antimicrobial activity while capped Ag NPs do [18,20]. Uncapped Ag NPs are unable to partition through the polysaccharide layers of the outer cell wall envelope, described in Figure 1, in order to bind to the cell membrane. This was verified by the TEM image shown in Figure 10a. SERS spectra obtained by forming the external colloid on the bacterial cell, bacteria(H_2_O)@AgNP, and mixing the cells with Ag NP, bacteria-AgNP, are shown in Figure 10b. The spectra are dramatically different. The spectrum of the cell-uncapped Ag NP mixture is similar to those shown inFigure 8b and Figure 9b and are due to the metabolites secreted by the cells. In contrast, the spectrum obtained for the externally formed colloid is dominated by flavin compounds.

**Figure 11:**
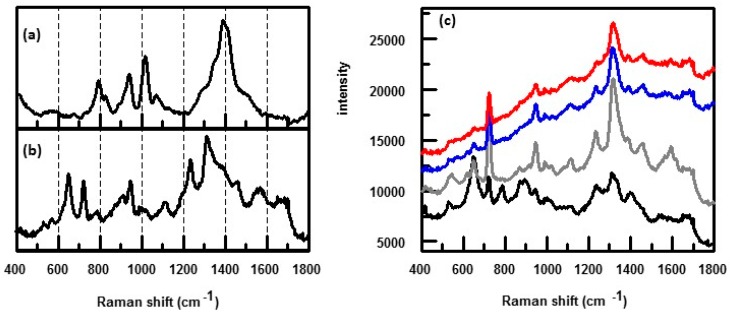
SERS spectra obtained using citrate generated Ag NPs [76]. Ag NPs were mixed with bacterial suspensions and filtered onto a 0.02 μm pore size ceramic filter. Spectra are: (**a**) citrate; (**b**) *Pseudomonas aeruginosa* P2; and (**c**) different ratios of Ag NPs to *E. coli* (top to bottom: 3:1, 2:1, 1:1, and 0.5:1 Ag NPs:*E. coli*). Spectra were obtained using 785 nm excitation. Reproduced with permission from the Elsevier.

Other groups have obtained SERS spectra by mixing capped/uncapped Ag NPs with bacteria [64,65,66,67,68,69,70,71,72,73,74,75,76]. The two sets of spectra differ. The difference is attributed to the manner in which the Ag NPs interact with the bacteria. Since uncapped Ag NPs do not bind directly to the bacteria, the spectral features are dominated by secretions from the cells. Capped Ag NPs can partition through the outer polysaccharide layers to bind to the cell membrane as shown in Figure 2 and Figure 3; however, an incubation time is required in order for this to occur. As the capped Ag NPs partition through the outer polysaccharide layers, they release the capping agent. This is shown by comparing spectra shown in Figure 11 [76]. Figure 11a shows the SERS spectrum of the citrate coating the Ag NPs. Figure 11b,c shows SERS spectra obtained for *P. aeruginosa* and *E. coli*, respectively, obtained by mixing equal volumes of citrate-generated Ag NPs and bacterial suspension. As can be seen, the citrate peaks are either eliminated or greatly reduced.

Citrate is a weakly bound capping agent on Ag/Au NPs and is therefore easily displaced by ligands that have a stronger affinity for those NPs [13]. Figure 11c show SERS spectra of *E. coli* obtained by varying the ratio of the bacteria suspension and the citrate-generated Ag NPs. The same peaks at the same relative intensities were observed for the 1:1, 2:1, and 3:1 Ag NPs:*E. coli* ratios. However, 2:1 and 3:1 Ag NPs:*E. coli* ratio spectra exhibit a broad fluorescence background. Both the decrease in the peak intensities and the fluorescence background with increasing amount of Ag NPs were indicative of damage to the cell walls of the bacteria resulting in leakage of the cellular contents. High fluorescence backgrounds have been observed when sodium borohydride was used to reduce silver ions adsorbed onto the bacteria [76]. Both a fluorescence background and a decrease in SERS signal of bacteria were observed when bacterial cells were washed with 1% NaCl solution [29]. Therefore, when cells lyse, the released cytoplasm contains sodium chloride which, in turn, causes a suppression of the SERS signal of the bacteria and a fluorescence background. Such damage is shown in the TEM images shown in Figure 3 [23].

The fact that the spectra obtained for 1:1, 2:1, and 3:1 Ag NPs:*E. coli* ratios exhibit the same relative peak intensities indicate that the Ag NPs have bound to the cell membrane. Unlike the spectra obtained by Zhou et al. [38] and Premasiri et al. [57], the spectra shown in Figure 11b,c are not dominated by peaks due to flavins and other secretions. The 1:1, 2:1, and 3:1 Ag NPs: *E. coli* spectra also show a small, narrow peak at ~1000 cm^−1^ that is attributed to the ring breathing mode of phenylalanine [78], an aromatic amino acid that is found in proteins. This peak is not observed in SERS spectra obtained using the external Ag colloid formation method (Figure 5b [31,34] and Figure 6b [38]). As shown in Figure 1, the cell membrane is a phospholipid bilayer with embedded proteins [8]. The 0.5:1 AgNPs: *E. coli* ratio spectrum, shown in Figure 11c, exhibits additional peaks not seen in the 1:1, 2:1, and 3:1 spectra. Some peaks in the 0.5:1 AgNPs: *E. coli* ratio spectrum are the same as that seen in the 1:1, 2:1, and 3:1 spectra. They do not appear to be due to the citrate capping agent. No fluorescence background is observed in the 0.5:1 ratio spectrum. The implications are that some Ag NPs are in contact with the cell membrane. However, other Ag NPs are trapped inside the cell wall envelope and peaks due to the polysaccharides, proteins, and lipids present in the cell wall envelope are observed [4]. Consequently, SERS spectra of bacteria obtained using capped Ag NPs contain contributions of metabolic secretions as well as structural components of the cell wall and cytoplasmic membrane.

### 2.3. Factors Affecting SERS Spectral Features Obtained for Bacteria

Besides the methodology used to obtain SERS spectra of bacteria, other factors influence the features that are observed in the spectra. As discussed above, spectra obtained using capped colloids are different from those obtained using uncapped colloids. This difference is attributed to the fact that capped Ag/Au NPs can partition through the outer layers of the cell envelope to reach the cytoplasmic membrane. Uncapped Ag/Au NPs cannot. As will be shown, the excitation wavelength used to obtain SERS spectra also influences what is observed as well as bacterial growth phase and stress/environmental factors.

#### 2.3.1. Effect of Laser Excitation Wavelength and Colloid Type

**Figure 12:**
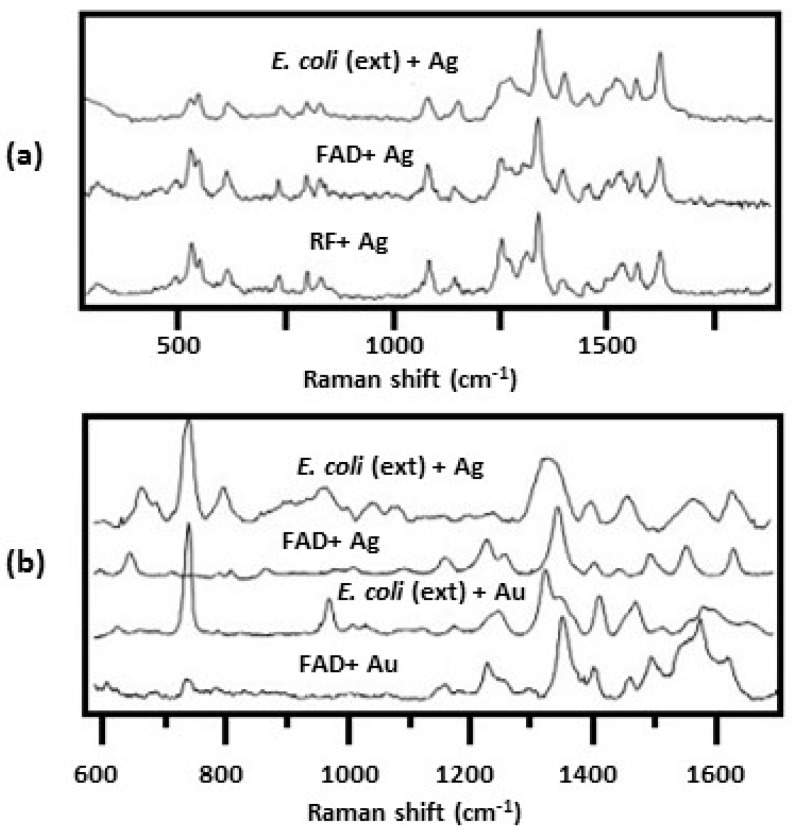
SERS spectra of *E. coli* coated with either an external Ag or Au colloid, FAD adsorbed on either Ag or Au, and RF adsorbed on Ag where spectra were obtained using: (**a**) 514 nm; and (**b**) 633 nm laser excitation [34]. Reproduced with permission from John Wiley and Sons.

Figure 12 shows SERS spectra obtained for *E. coli*, flavin adenine dinucleotide (FAD), and riboflavin (RF) obtained on either Ag or Au colloid using either 514 or 633 nm laser excitation [34]. For *E. coli*, the colloid was formed externally on the cells by using sodium borohydride to reduce either AgNO_3_ or HAuCl_4_. It can be seen that the observed spectral features were dependent upon excitation wavelength used as well as the colloid type. Using 514 nm laser excitation, the SERS spectrum of *E. coli* is very similar to that of FAD and RF, Figure 12a. Both FAD and RF have a strong absorption at 450 nm with a tail extending to 520 nm. With 514 nm excitation, both FAD and RF are in a preresonance condition which results in further enhancement of the signal. Consequently, the flavin spectrum dominates the SERS spectrum of *E. coli* masking the spectral contributions of other constituents that make up the cell envelope.

By going to 633 nm laser excitation, the flavin preresonance is turned off and the SERS spectrum of *E. coli* showed additional peaks besides those attributed to the flavins, Figure 12b [34]. Going to an external Au colloid showed even more peaks that were due to other cell components, Figure 12b. The main features shown using 633 nm laser excitation were strong peaks at 735 and 1330 cm^−1^ that are due to adenine. This adenine could be due to FAD or other adenine bearing molecules such as nicotinamide adenine dinucleotide (NAD), ATP, DNA, etc. The results summarized in Figure 12 demonstrate another one of the strengths of the SERS technique in obtaining spectra of bacteria: features that are emphasized in the spectra can be controlled by simply changing the colloid used or the excitation wavelength.

#### 2.3.2. Bacterial Growth Phase

Spectral features observed in SERS spectra are dependent upon the growth phase of the bacteria [55,71]. One study placed harvested bacteria on Ag/AAO substrates [55]. Another study mixed bacteria with hydroxylamine-generated Ag NPs that were then placed on the surface of CaF_2_ slides [71]. Because both studies used uncapped Ag NPs, SERS spectra were dominated by cell metabolic secretions, discussed in Section 2.2.2 and Section 2.2.3.

**Figure 13:**
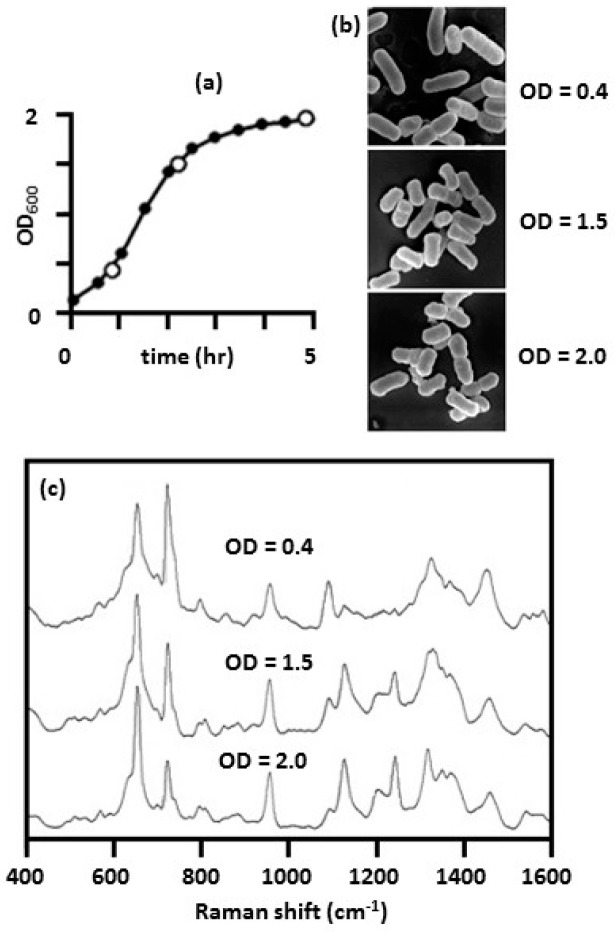
SERS spectra of *E. coli* obtained at different growth phases [55]: (**a**) plot of optical density at 600 nm (OD_600_) as a function of growth time, where cells were harvested at OD_600_ of 0.4, 1.5, and 2.0 (open circles); (**b**) SEM images of harvested bacteria; and (**c**) SERS spectra of harvested bacteria, where spectra were obtained by placing the bacteria on an Ag/AAO substrate and using 632.8 nm laser excitation. Reproduced with permission from the PLOS.

Figure 13 summarizes results obtained for *E. coli* on Ag/AAO substrates [55]. The density of *E. coli* in broth culture was quantified by measuring the turbidity or optical density at 600 nm (OD_600_). Figure 13a shows a plot of OD_600_ as a function of growth time. Three distinctive growth phases were observed at OD_600_ 0.4, 1.5, and 2.0 corresponding to bacterial growth at the beginning of the exponential phase, the middle to late exponential phase, and the stationary phase, respectively. Cells were harvested at these three growth phases. After washing, SEM images were taken of the cells, Figure 13b, and SERS spectra were measured, Figure 13c. The SEM images, in Figure 13b, revealed a decrease in the aspect ratio of the rod-like bacteria indicating a “shortening” of the *E. coli* cells as the bacteria growth approached a maximum. Significant changes were observed in the SERS spectra as shown in Figure 13c. The intensity of peaks at 655, 1130, 1219, and 1245 cm^−1^ were observed to progressively increase as the bacteria moved from the exponential phase to the stationary phase. Over the same period, the intensities of the peaks at 725 and 1095 cm^−1^ gradually decreased. Because of the nature of the SERS substrates used, the spectral features observed in the SERS spectra are due to the metabolic secretions of the cells. The intensity changes observed in the SERS spectra, and the morphology changes observed in the SEM images, demonstrate the highly dynamic character of the bacteria cell wall and metabolites present on the outer surface of the cell wall [55,71].

#### 2.3.3. Stress/Environmental Factors

The composition of secretions, carbohydrates, proteins, and lipids in the external cell envelope of bacteria results from environmentally induced differential gene expression and is indicative of how a cell responds to, and interacts with its environment. A normal Raman study of *E. coli* and *S. epidermidis* was done in which the cells were inactivated by different chemicals and stress conditions including starvation and high temperature [79]. Significant changes were observed in the spectra of treated cells in comparison with cell samples that had not undergone treatment.

**Figure 14:**
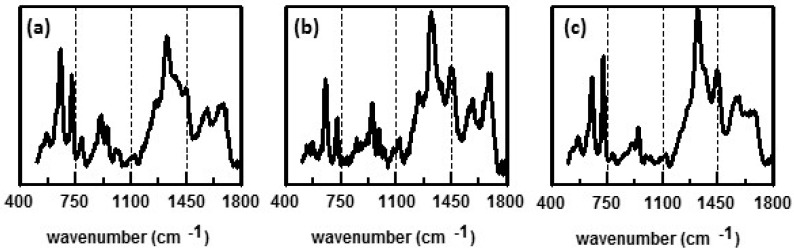
Preliminary SERS results obtained for *E. coli* subjected to different temperatures: (**a**) 4 °C for 245 min; (**b**) 37 °C (temperature the cells were cultured); and (**c**) 43 °C for 64 min. *E. coli* samples were mixed with citrate-generated Ag NPs and filtered onto a ceramic substrate [76]. SERS spectra were obtained using 785 nm laser excitation.

Few such studies have been done using SERS. Preliminary experiments stressing *E. coli* using heat/cold treatment have been done. Cells were grown at 37 °C. A sample of these cells were mixed with citrate-generated Ag NPs. Two samples of the cells were then subjected to temperatures of 4 and 45 °C for 245 and 64 min, respectively. Afterwards they were mixed with citrate-generated Ag NPs. After incubating overnight, all three samples were filtered onto a ceramic filter as described above [76]. SERS spectra are shown in Figure 14. Significant differences can be seen in the spectra, particularly in the region between 1200 and 1700 cm^−1^. While preliminary, these results demonstrate that SERS may be used to directly monitor changes in the bacterial cell membrane and metabolism as a result of stress.

SERS has been used to assess a bacteria’s susceptibility to an antibiotic [54,55]. In these investigations, *E. coli* and *S. aureus* were treated with antibiotics that targeted the cell wall (ampicillin, oxacillin, vancomycin, cefotaxime, gentamicin, and tetracycline). Antibiotic-treated cells were placed on Ag/AAO substrates that measure cell secretions as discussed in Section 2.2.2. It was shown that discernable SERS changes in response to antibiotics that interfere with general bacterial protein synthesis was not evident until after 9–12 h of antibiotic treatment [55]. This was because cell wall integrity could be maintained for a long time even in the absence of new protein synthesis.

### 2.4. Principal Component Analysis

Principal component analysis (PCA) is a statistical procedure for identifying a smaller number of uncorrelated variables, called “principal components” to reduce the dimensionality for a large set of data [80,81]. The goal of principal component analysis is to explain the maximum amount of variance with the fewest number of principal components. PCA has been applied to analyze SERS spectral data obtained for bacteria. In order to understand the significance of the correlations, it is necessary to know the nature of the chemical species responsible for the peaks observed in the SERS spectra. This section discusses peak assignments as well as the use of PCA of bacterial SERS spectra.

#### 2.4.1. Peak Assignments of Bacterial Spectral Features

Determining the chemical species responsible for the peaks observed in the SERS spectra of bacteria is challenging. There is significant overlap of peaks due to proteins, carbohydrates, lipids, flavins, purines, and pyrimidines. As was discussed in the previous sections, the spectral features observed are dependent upon the kind of SERS substrates used to obtain the spectra, whether capped or uncapped colloids were used, the laser excitation, the bacterial growth phase, and environmental conditions. For SERS obtained using the external colloid formation method, the isoalloxazine fused ring system of flavins serves as an efficient center of nucleation of silver colloids in or on the cell wall ensuring its proximity to the SERS-active sites [34]. Consequently, the spectra obtained using this method are dominated by features due to flavins. Spectra obtained by either placing bacteria on SERS-active nanoparticles immobilized on a surface [57] or mixing bacteria with uncapped Ag/Au NPs [38] are dominated by spectral features due to metabolic secretions. For both of these methods, the Ag/Au NPs are unable to partition through the outer polysaccharide layers to bind to the cytoplasmic membrane. In contrast, spectra obtained by mixing bacteria with capped Ag/Au NPs exhibit spectral features due to both metabolic secretions and the cell wall. This is because capped Ag/Au NPs can partition through the outer cell envelope and can bind to the cytoplasmic membrane.

**Figure 15:**
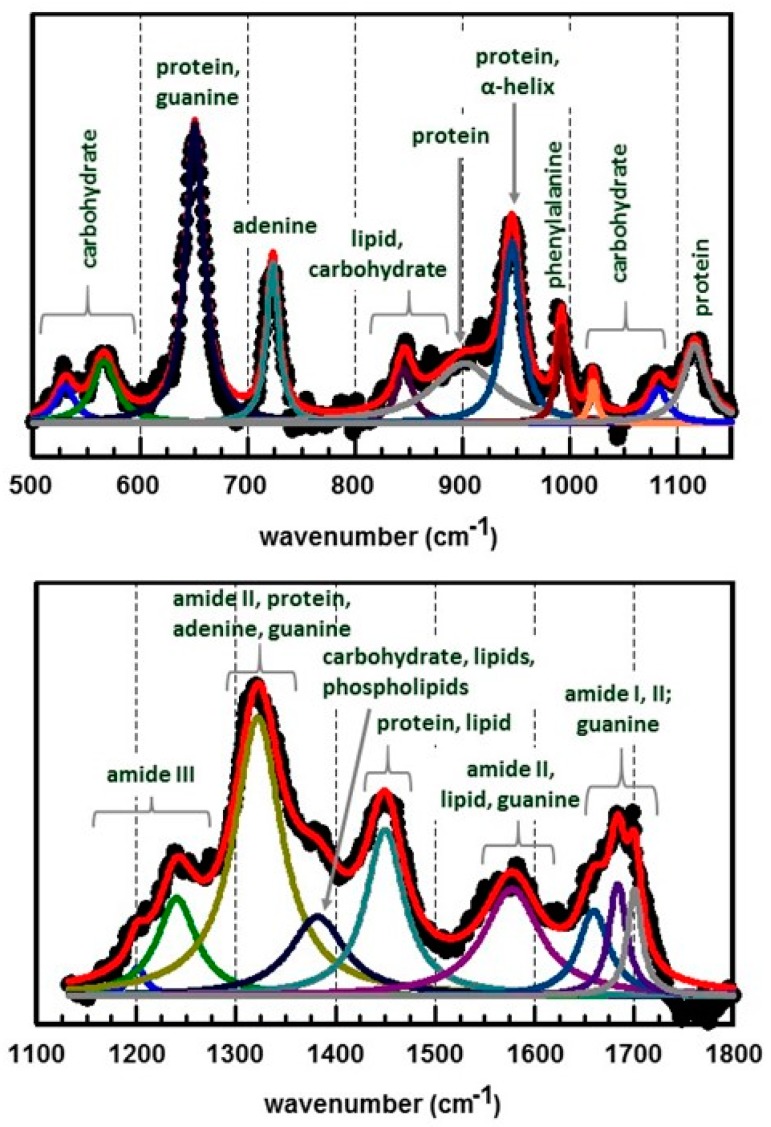
Deconvoluted SERS spectrum of *E. coli* (full spectrum shown in Figure 14b) obtained by mixing citrate generated Ag NPs with the bacterial suspension and filtering onto a 0.02 μm pore size ceramic filter [76]. Spectrum was obtained using 785 nm excitation. Data points are shown as black, filled circles. The red line is the summation of all the Lorentzian peaks. Peak assignments are shown.

The spectral bands observed in the SERS spectrum obtained for *E. coli*, Figure 14b, were deconvoluted into their component peaks. The line shape of a SERS peak was described by a simple Lorentzian [82]. Therefore, a spectral band, *S*(*ν*), consisting of M Lorentzian peaks is represented by the following expression:

(1) $$S\left(\nu \right)=\sum_{i=1}^{M}\frac{I_{Oi}\sigma_{i}^{2}}{\sigma_{i}^{2}+{\left(\nu -\nu_{Oi}\right)}^{2}}$$

where each SERS peak, *i*, is characterized by a center position, *ν_oi_*, with a maximum intensity, *I_oi_*, and standard deviation, *σ_i_*. Results of these deconvolutions are summarized in Figure 15. This spectrum was obtained by mixing citrate-generated Ag NPs with the bacteria. Consequently, the spectrum contains contributions due to both metabolic secretions and the cytoplasmic membrane. Peak assignments are shown [30,41,66,73,78,83,84,85,86,87,88,89].

#### 2.4.2. Summary of PCA Results Obtained for Bacteria

As summarized in Section 2.2, three general methods have been used to obtain SERS spectra of bacteria. Method 1 creates an external Ag/Au colloid on the bacteria. In Method 2, the bacteria are placed on top of a SERS-active surface. Method 3 mixes Ag/Au colloid with the bacteria and then places the mixture on top of a flat surface. PCA has been employed to evaluate SERS spectra obtained for all three methods.

Chen et al. [35] obtained SERS spectra of *L. monocytogenes*, *E. coli*, methicillin resistant *S. aureus* (MRSA), *P. aeruginosa*, and *L. innocua* using the external colloid method. Using PCA, they were able to discriminate these species of bacteria. Colniță et al. [39] also obtained SERS spectra of *L. casei* and *L. monocytogenes* using the external colloid method. According to the PC loading plots, the spectral differenced that contributed to the discrimination of the two species were related to the 731 cm^−1^ adenine vibration band and the SERS band assigned to CC, CO, COH deformations in carbohydrates or C-C stretching in lipids, CH deformations in proteins, and CH_2_ deformations of saturated lipids.

**Figure 16:**
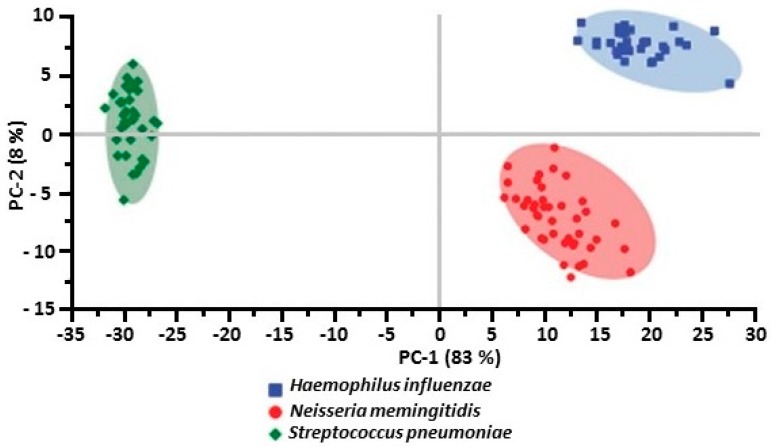
The comparison of the calculated PCA analysis for three bacteria: *S. pneumoniae* (green diamonds), *H. influenzae* (blue squares), and *N. meningitidis* (red circles) [51]. Reproduced with permission from the Royal Society of Chemistry.

Kamińska et al. [51] obtained SERS spectra of bacterial meningitis pathogens using method two. They used PCA to evaluate the spectral differences among the clinical samples infected by *N. meningitidis*, *S. pneumoniae*, and *H. influenza* along with the normal (control) type and to develop models allowing the simultaneous discrimination and classification of these three meningitis pathogens in clinical samples. In PCA, variables with high loading values are the most important for diagnostic purposes. The most important variations among these three meningitis pathogens were found to be associated with peaks at 644, 738, 744, and 1330 cm^−1^ that corresponded to the main compounds of bacterial cells. The PCA evaluation performed for these three pathogens in the 500–1600 cm^−1^ fingerprint region gave the sum of PC-1 and PC-2 equal to 91% of the total variance. Figure 16 show a comparison of the calculated PCA analysis for the three bacteria.

Stephen et al. [46] and Patel et al. [90] also obtained SERS spectra of bacteria by placing them on the surface of a SERS-active substrate (method two). Stephen et al. [46] used PCA, in conjunction with linear discrimination analysis (LDA), to classify fourteen strains of *Arthobacter* with 97% accuracy. Patel et al. [90] applied PCA to the first and second derivative SERS spectra of bacteria. First derivative spectra avoided contributions resulting from fluctuations in spectral background but were still sensitive to SERS vibrational intensity fluctuations. Second derivative SERS spectra also minimized background variability and further reduced sensitivity to intensity fluctuations. They showed that a second derivative based clustering approach, combined with the reproducibility provided by their method of obtaining SERS spectra, resulted in excellent species and strain level clusters for bacterial identification in a group of closely related bacteria.

Cam et al. [74] obtained SERS spectra of mixtures of three different but related bacterial species: *Shigella sonnei*, *Proteus vulgaris*, and *Erwinia amylovara*. These three bacterial species belong to the same family (Enterobacteriaciae). They also prepared mixtures of three strains of *Escherischia coli*. To obtain SERS spectra, the bacterial mixtures were mixed with citrate-generated Ag NPs and the resultant suspension was spotted on a CaF_2_ surface (method three). PCA analysis showed that it was possible to identify the composition of bacteria in a mixture.

**Figure 17:**
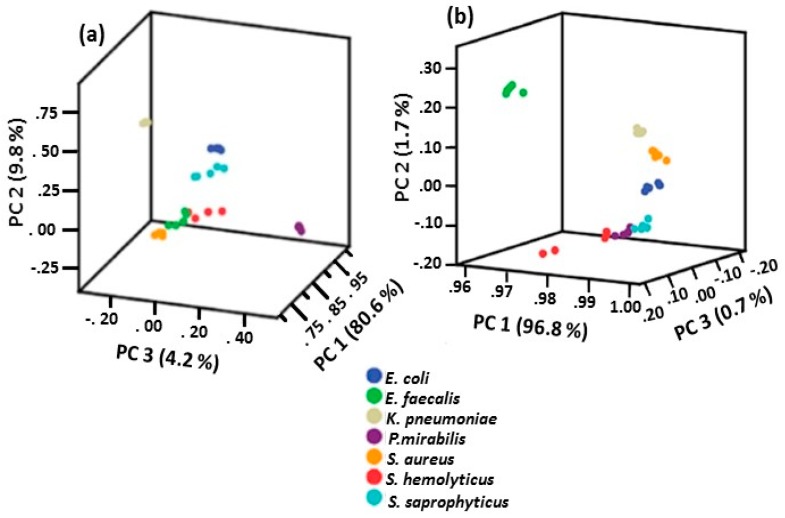
PCA plots of bacteria at: (**a**) 1 h; and (**b**) 24 h of incubation [71]. Reproduced with permission from Springer.

Avci et al. [71] obtained SERS spectra of urinary tract infection (UTI) pathogens by mixing the bacteria with hydroxylamine-generated Ag NPs and placing the suspension on a CaF_2_ surface (method three). Knowing that bacteria respond to their environment by changing their metabolic profiles and composition of their cell walls and that the composition of the cell wall is dependent upon growth phase, SERS spectra of UTI–related bacteria were obtained at different growth phases. The spectral pattern differences among the seven bacterial species was at the highest level after 1 h of incubation and decreased with increasing incubation time. The increase in spectral differences within the 1 h incubation time reflected the metabolism differences among the different bacteria species. PCA was performed on the bacteria spectra obtained at different time points. Results are shown in Figure 17. This study showed that all species can be differentiated regardless of their growth phases using PCA. As shown in Figure 17, the percent of first principal component, which had the largest possible variance and explained most of the variance, increased from 80.6 to 96.8 in 24 h. Generally, 24 h grown bacteria species have been used for identification and discrimination of bacteria using SERS. The results summarized in Figure 17 show that there is no need for 24 h incubation and only 1 h is sufficient for discrimination of bacterial species.

## 3. Conclusions

In this communication, the nature of the interaction of Ag/Au NPs with bacteria was discussed as well as how these interactions affect what is observed in the SERS spectra obtained for bacteria. It was shown that the interpretation of SERS spectra obtained for bacteria is complicated. The features observed in the spectra are dependent upon the following:

- Method used to obtain SERS spectra, i.e., internal/external colloid formation, placement of bacterial suspension on top of a SERS-active surface, or mixing Ag/Au NPs with bacteria
- Use of capped or uncapped Ag/Au NPs when using the mixing protocol
- Laser excitation wavelength used to generate the spectra
- Growth phase of the bacteria
- Bacterial interactions with the environment

Consequently, the effect these factors have on bacteria need to be understood to correctly interpret the spectral results.

As was discussed, protocols involving the formation of an external colloid and mixing with capped Ag/Au NPs provided information on cell secretions and cell wall composition. Mixing with uncapped Ag/Au NPs and placing bacteria on a SERS active surface provided information on cell secretions. Use of 514 nm laser excitation resulted in spectra that are dominated by peaks due to flavins. Consequently, the type of substrates used as well as the laser excitation can control what is observed in a spectrum. This makes SERS a very powerful tool to study bacterial species. It was shown that SERS can be used to identify/discriminate bacterial species/strains, monitor quorum sensing between bacteria and germination of spores, and ascertain susceptibility to antibiotics. SERS of bacteria also has the potential to monitor the impacts of climate change as the bacterial cell wall and the metabolites present in the outer surface of the cell wall are dynamic and will respond to such environmental factors as temperature, salinity, and pH.

## Figures and Tables

**Table 1:** Summary of bacteria detected using either external wall or internal colloids that were discussed in this review.

SERS Method	SERS Substrate	Bacterial Species and References
external wall colloid	borohydride used to reduce Ag on the cell wall; treated cells placed on a microscope slide	*Escherichia coli* [30,31,32,34,35]
		*Bacillus megaterium* [30,31,32,34]
		*Acinetobacter calcoaceticus* [31,32]
		*Pseudomonas aeruginosa* [31,32,35]
		*Listeria innocua* [35]
		*Listeria monocytogenes* [35]
		*Staphylococcus aureus* [35]
external wall colloid	borohydride used to reduce Au on the cell wall; treated cells placed on a glass slide	*Escherichia coli* [34]
external wall colloid	borohydride used to reduce Ag on the cell wall; treated cells placed on an Al SEM sample stub	*Escherichia coli* [33]
		*Bacillus subtilis* [33]
external wall colloid	hydroxylamine used to reduce Ag on the cell wall; treated cells placed on a glass slide or a poly-l-lysine coated glass slide	*Escherichia coli* [38,39,40]
		*Staphylococcus epidermidis* [38,39]
		*Aeromonas* [39,40]
		*Pseudomonas aeruginosa* [39]
		*Proteus mirabilis* [39]
		*Lactobacillus casei* [39,40]
		*Morganella morganii* [40]
		*Listeria monocytogenes* [40]
		*Lactococcus lactis* [40]
external wall colloid	hydroxylamine used to reduce Ag on the cell wall; treated cells placed on a MgF_2_ slide	*Lactobacillus casei* [42]
		*Listeria monocytogenes* [42]
internal colloid	borohydride used to reduce Ag inside the cell; treated cells placed on a glass slide	*Escherichia coli* [33,34]
		*Acinetobacter calcoaceticus* [33,34]

**Table 2:** Summary of bacteria detected by placing bacteria directly on a SERS-active surface that were discussed in this review.

SERS Substrate	Bacterial Species and Reference
roughened Au coated glass slide	*Anthrobacter* [46]
Ag NPs (H_2_ reduction of Ag_2_O) immobilized on Ag mirrored glass slide	*Bacillus subtilis* [47]
Au@pNIPAM hydrogels; mesostructured Au@TiO_2_; micropatterned Au@SiO_2_ supercrystal arrays	*Pseudomonas aeruginosa* [48]
Ag NPs formed by the bacteria on a Ag/AgCl solid interface	*Shewanella oneidensis* [49]
thin Au film deposited onto a PPX-Cl surface using thermal evaporation	*Escherichia coli* [50]
Ag/Au film on 3 and 0.3 μm pore size, polycarbonate membranes	*Haemoplilus influenzae* [51]
	*Neisseria meningitidis* [51]
	*Streptococcus pneumoniae* [51]
filter made of borohydride generated Au NPs embedded in mesoporous silica	*Staphylococcus aureus* [52]
	*Staphylococcus aureus* [54]
	*Enterococcus feacalis* [54]
	*Listeria monocytogen* [54]
	*Escherichia coli* [54]
	*Klebsiella pneumoniae* [54]
	*Serratia marcescens* [54]
	*Mycobacterium tuberculosis* [54]
	*Mycobacterium gordonae* [54]
electrodeposited Ag NPs in AAO channels [53]	
electrodeposited Ag NPs in AAO channels [53]; coat array with vancomycin	*Escherichia coli* [55]
	*Enterococcus feacalis* [55]
	*Lactobacillus plantarum* [5]
borohydride generated Au NP-covered SiO_2_ substrate	*Escherichia coli* [56,57]
	*Bacillus cereus* [56]
	*Bacillus anthracis* [56,57]
	*Bacillus subtilis* [56]
	*Bacillus thuringiensis* [56]
	*Salmonella typhimurium* [56]
	*Staphylococcus aureus* [57]
	*Streptococcus agalactiae* [57]
	*Streptococcus pneumonia* [57]
	*Pseudomonas aeruginosa* [57]
	*Pseudomonas putida* [57]
	*Enterococcus faecium* [57]
	*Enterococcus feacalis* [57]
	*Acinetobacter baumannii* [57]
4-mercaptophenylboronic acid functionalized Ag dendrites	*Salmonella enterica* [59]
Immunomagnetic separation followed by concentration on a SERS substrate	*Listeria innocua* [60]

**Table 3:** Summary of bacteria detected by mixing colloids with bacteria that were discussed in this review.

SERS Substrate	Bacterial Species and Reference
hydroxylamine-generated Ag NPs; mixture placed on a glass slide	*Escherichia coli* [38,64]
hydroxylamine-generated Ag NPs; mixture placed in a glass capillary tube	*Bacillus thuringiensis* [65]
citrate capped, borohydride-generated Ag NPs; mixture placed in a glass capillary tube	*Bacillus thuringiensis* [65]
borohydride-generated Ag NPs; SERS obtained of mixture	*Escherichia coli* [66,67,68]
	*Pseudomonas aeruginosa* [66,67,68]
	Artic psychro-active marine bacteria [66]
	*Salmonella typhimurium* [68]
borohydride-generated Ag NPs; mixture placed on CaF_2_ slides	*Escherichia coli* [69]
	*Bacillus megaterium* [69]
hydroxylamine-generated Ag NPs; mixture placed on CaF_2_ slides	*Escherichia coli* [71]
	*Enterococcus faecalis* [71]
	*Staphylococcus aureus* [71]
	*Staphylococcus saprophyticus* [71]
	*Klebsiella pneumoniae* [71]
citrate-generated Ag NPs; mixture placed on CaF_2_ slides	*Escherichia coli* [69,72,73,74]
	*Bacillus megaterium* [69,73]
	*Shigella sonnei* [72,74]
	*Erwinia amylovara* [72,74]
	*Proteus vulgaris* [72,74]
	*Staphylacoccus cohnii* [72]
	*Staphylacoccus aureus* [72]
citrate-generated Ag NPs; mixture placed on glass slides	*Escherichia coli* [70]
	*Staphylacoccus cohnii* [70]
Ag NPs conjugated with synthetic peptides (pgSERS probes)	*Escherichia coli* [75]
citrate-generated Ag NPs; mixture filtered onto a ceramic filter	*Escherichia coli* [76]
	*Shewanella putrefaciens* [76]
	*Pseudomonas aeruginosa* [76]
